# Comparative study of young shoots and the mature red headed cabbage as antioxidant food resources with antiproliferative effect on prostate cancer cells

**DOI:** 10.1039/d0ra07861a

**Published:** 2020-11-26

**Authors:** Mariola Drozdowska, Teresa Leszczyńska, Aneta Koronowicz, Ewelina Piasna-Słupecka, Kinga Dziadek

**Affiliations:** Department of Human Nutrition and Dietetics, Faculty of Food Technology, University of Agriculture in Krakow 122 Balicka St. 30-149 Krakow Poland mariola.drozdowska@urk.edu.pl

## Abstract

The increasing knowledge on health benefit properties of plant origin food ingredients supports recommendations for the use of edible plants in the prevention of diet related diseases, including cancer. The beneficial effects of young shoots of red cabbage can be attributed to their mixture of phytochemicals possessing antioxidant and potential anticancer activity. The objective of this study was to compare the content of bioactive compounds, including HPLC analysis of polyphenols and antioxidant activity of young shoots of red cabbage and the vegetable at full maturity. The content of vitamin C and polyphenols in juices obtained from young shoots and the mature vegetable were also determined. The other aim of this study was to confirm the hypothesis that juice of young shoots more effectively, compared to juice of the mature vegetable, reduces the proliferation of prostate cancer cell lines DU145 and LNCaP *in vitro*. A significantly higher content of vitamin C and carotenoids, as well as a higher antioxidant activity were found in edible young shoots in comparison to the mature vegetable. In addition, studies have shown higher amount of vitamin C in the juice of young shoots than in the juice of the mature vegetable and similar content of polyphenolic compounds. The level of total polyphenol content in the studied plant samples did not differ significantly. Flavonoids were the main polyphenols in young shoots and juice obtained from them, while phenolic acids were dominant in the mature vegetable and in juice obtained from it. The juice of young shoots has shown stronger *in vitro* anti-proliferation effect against prostate cancer cells than juice of the mature vegetable.

## Abstract 2

Young shoots of red cabbage could be a good source of phytochemicals with potential anticancer activity.

## Graphical abstract

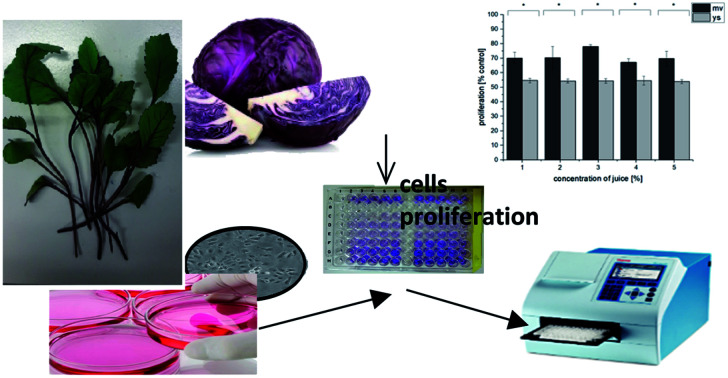

## 1. Introduction

The widespread threat of diet-related diseases has led to a conscious search for health benefiting food, including preventive and functional food. Fruits and vegetables are an important part of the human diet and provide valuable nutrients. Identifying and assessing the health properties of plant bioactive compounds can help understand the close relationship between diet and non-communicable diseases. According to WHO recommendations, 400 g of fruit and vegetables should be eaten each day to supply the right amount of nutrients that are essential for good health. The beneficial effects of plants have been attributed to compounds with *e.g.* antioxidant activity, which provide bioactive mechanisms to reduce free radical induced oxidative stress. In the case of food of plant origin, primarily vitamins, carotenoids and polyphenolic compounds show antioxidant properties.^1^ Antioxidants are capable of scavenging free radicals, inhibiting pro-oxidative enzymes and protecting major biological macromolecules (DNA, protein and lipid) in cells and tissues.^2^

Cruciferous vegetables are a valuable source of protective phytochemicals with health benefits. The direct antioxidant and anticancer properties of cabbages are mainly due to the content of vitamin C, carotenoids, polyphenols (such as anthocyanins), and the most characteristic compounds – glucosinolates.^3^ The mechanisms of the anticancer action of glucosinolates degradation products are based on the ability to modulate the expression of enzymes of phase I and II detoxification, the prevention of DNA damage in the cell as well as cell cycle regulation and apoptosis.^4^ In European folk medicine, cabbage leaves were used to treat acute inflammation. Leaves of raw cabbage may be placed around the affected area to reduce pain. Another interesting fact is effective in relieving painfully engorged breasts in breastfeeding women.^5^

Red cabbage (*Brassica oleracea* var. *capitata* f. *rubra*) is a popular cruciferous vegetable all over the world and is eaten raw and after technological treatment. *Brassicas* can be consumed in different forms such as a salad, fresh, cooked, fried, baked or fermented. This vegetable is attractive for consumers because of its dietetic and taste values and also its characteristic red color, which it owes to flavonoids, specifically anthocyanins.^6^ Red cabbage contains a range of different bioactive compounds which have a beneficial impact on human health and help in protecting against cancer agents encountered every day in our environment.^7^ In recent years, cruciferous vegetables in a germinating stage such as sprouts have been appeared on the market.^8^ It is known that changes occur during germination in the nutritional profile of sprouts where a higher content of phenolic compounds is observed in this phase.^9^ Our previously research indicated that young shoots of red cabbage are a better source of protein and selected minerals as well as glucosinolates in comparison to the vegetable at full maturity.^10^ Edible sprouts and young shoots of vegetables can be produced without extensive product development, new equipment or costly marketing.

According to available literature, there is a lack of data on the content and identification of bioactive compounds in the young shoots of vegetables, including red headed cabbage and the influence of these compounds on the proliferation of prostate cancer cell lines *in vitro*. The objective of this research was to determine and identify the bioactive compounds, especially vitamin C, carotenoids and polyphenols, as well as the examination of the antioxidant activity of young shoots of red headed cabbage and the mature vegetable. The other aim of this study was to confirm the hypothesis that young shoots have a higher antioxidant potential than the vegetable at full maturity. Additionally, the content of vitamin C and polyphenols in juices of young shoots and the mature vegetable were determined. Other hypothesis was also checked-whether juice of young shoots more effective reduces the proliferation of prostate tumor cells *in vitro* in comparison to juice of the mature vegetable.

## 2. Material and methods

### 2.1. Plant material

The analyzed materials were ready to eat, fourteen-day young shoots of red cabbage and the vegetable at full maturity (polish variety Haco). The young shoots were grown in sowing boxes filled with a standard garden substrate in late May. The same seedlings were planted into the ground. Red headed cabbage was sown on brown soil with pH 6.5, salinity 0.57 g L^−1^ NaCl, NH_4_ 3.5 mg L^−1^, NO_3_ 52.5 mg L^−1^, P 187 mg L^−1^, K 187 mg L^−1^, Ca 1324 mg L^−1^, Mg 188.45 mg L^−1^. The young shoots were collected in May 2018 and 2019. The vegetable at full maturity were collected in October 2018 and 2019. The average temperature (°C) and total monthly rainfall (mm) during 5 month vegetables growth in 2018 was in: June – 18.9 and 72; July – 19.9 and 142; August – 20.6 and 71; September – 15.6 and 43; October – 10.4 and 52, respectively. The young shoots were analyzed in their entirety. Samples of the mature vegetables were prepared by cutting different-sized heads. The fresh material was used to prepare juices (by squeezing in a juicer). The fresh vegetables were transferred to plastic plates and frozen at −80 °C and placed in a freeze dryer for 24 h [Christ Alpha 1–4 (Osterode am Harz, Germany)]. After freeze drying, the samples were closed in a plastic bag to protect them from moisture and stored in a freezer (−20 °C) for further analysis. Additionally, the fresh plants were used to prepare methanolic extracts and determine the level of vitamin C.

### 2.2. Extracts preparation

One gram of fresh samples was weighed and added to 80 mL of 70% methanol acidified HCl in order to measure the total polyphenol and anthocyanin content as well as antioxidant activity. Lyophilized plant material was extracted with 40 mL of 0.1% formic acid dissolved in 70% HPLC grade methanol (w/v) in order to identify the polyphenolic compounds with HPLC. Then, the samples were shaken at room temperature for 2 h (Elpin Plus, water bath shaker type 357, Lubawa, Poland) and centrifuged at 1500 rpm for 15 min (centrifuge type MPW-340, Warsaw, Poland). Then the supernatant was decanted and stored at −20 °C until further analysis.

### 2.3. Determination of selected bioactive compounds

The content of vitamin C, as a sum of ascorbic and dehydroascorbic acid, in the examined material, was determined by Tillmans method in Pijanowski's modification.^11^ This assay is based on reduction of dehydroascorbic acid to ascorbic with use sodium sulfide. The total ascorbic acid is determine by 2,6-dichlorophenolindophenol titration. An acetone–hexane mixture (4 : 6 v/v) was used for extraction of carotenoids from the lyophilized samples according to the PN-EN 12136 : 2000. The concentration of total carotenoids was measured spectrophotometrically at 450 nm (spectrophotometer UV-1800, RayLeigh, Beijing Beifen-Ruili Analytical Instrument Co., Ltd, Beijing, China). The content of total polyphenols in the extracts was estimated spectrophotometrically at 760 nm (spectrophotometer, as above) using the Folin–Ciocialteu reagent (Sigma Aldrich, st. Louis, MO, USA).^12^ The results were expressed as chlorogenic acid equivalents (CGA) in mg CGA per 100 g of sample. The content of total anthocyanins was studied spectrophotometrically in methanol extracts by the pH differential methods, using a potassium chloride buffer, pH 1.0 (0.025 M) and sodium acetate buffer, pH 4.5 (0.4 M).^13^ The absorbance was measured at 510 nm and 700 nm (spectrophotometer, as above).

### 2.4. HPLC analysis of polyphenols

The HPLC analysis of polyphenols was conducted using the Prominence-i LC-2030C 3D Plus system (Shimadzu, Kyoto, Japan) equipped with a diode array detector (DAD). The separation was performed on the Luna Omega 5 μm Polar C18, 100 A, 250 × 10 mm column (Phenomenex, California, USA) at 40 °C. The mobile phase was a mixture of two eluents: A – 0.1% formic acid in water (v/v) and B – 0.1% formic acid in methanol (v/v). The flow rate of the mobile phase was 1.2 mL min^−1^. The analysis was carried out with the following gradient conditions: from 20% to 40% B in 10 min, 40% B for 10 min, from 40% to 50% B in 10 min, from 50% to 60% B in 5 min, 60% B for 5 min, from 60 to 70% B in 5 min, from 70% to 90% B in 5 min, 90% B for 5 min, from 90% to 20% B (the initial condition) in 1 min and 20% B for 4 min, resulting in a total run time of 60 min. The injection volume was 20 μL. The detection of 4-hydroxybenzoic acid, myricetin, luteolin and isorhamnetin was done at 254 nm, rutin at 256 nm, vanillic acid at 260 nm, kaempferol at 264 nm, apigenin at 267 nm, gallic acid at 271 nm, syringic acid at 274 nm, catechin and epicatechin at 278 nm, naringin at 283 nm, hesperidin at 284 nm, *p*-coumaric acid at 310 nm, caffeic acid, ferulic acid and sinapinic acid at 323 nm as well as chlorogenic acid at 326 nm. Quantitative determinations were carried out using calibration curve of the standards. Individual stock standard solutions of each phenolic compound were prepared in 0.1% formic acid in 70% methanol at a concentration of 100 mg L^−1^. The calibration curves were made by using the working standard solutions which were prepared by mixing suitable volumes of each stock standard solutions, to give concentrations ranging from 0.5 to 4.0 mg L^−1^. All working standard solutions and studied samples were filtered by a 0.22 μm pore size membrane filter. The data were integrated and analyzed using the LabSolutions software ver. 5.93 (Shimadzu Corporation, Kyoto, Japan).

### 2.5. Determination of antioxidant activity

The antioxidative activity of the samples was measured using three methods: ABTS˙* (2,2′-azinobis-(3-ethylbenzothiazoline-6-sulfonic acid)), DPPH (2,2-diphenyl-1-picrylhydrazyl) and FRAP (ferric reducing antioxidant power). The assays were according to the procedure reported by Dziadek *et al.*^14^ The ABTS˙* method involved ability of samples to extinguish ABTS˙* free radical, which has been reduced by the antioxidants contained in the analyzed product. The antioxidant compounds present in the samples convert DPPH radical to a more stable DPPH˙ molecular product by donating an electron or a hydrogen atom. The FRAP assay was based on the reducing power of antioxidants which reduce the ferric ion (Fe^3+^) to the ferrous ion (Fe^2+^). All the methods require a UV-Vis spectrophotometer. The results were calculated based on the calibration curve and expressed as micromoles of Trolox equivalent per gram of sample (TEAC).

### 2.6. Cell proliferation

The human prostate carcinoma DU145 (not detectably hormone sensitive, ATCC®HTB-81™) and LNCaP cell lines (androgen receptor, positive; estrogen receptor, positive; ATCC®CRL-1740™) were purchased from the American Type Culture Collections. The human normal prostate PNT-2 cell line was purchased from HPA Culture Collections (Sigma-Aldrich, MO, USA). Cells were cultured in appropriate RPMI 1640, medium (Sigma-Aldrich, MO, USA) according to the ATCC protocol with an addition of 10% FBS (Sigma-Aldrich, MO, USA). Cells were seeded on 96-well plates (8 × 10^4^ cells per well). 24 h after seeding, growth medium was replaced with a medium containing juices of young shoots or the vegetable at full maturity. Percentage dilution of juices were prepared in the appropriate cell culture medium and added to the wells on growth plates. The final applied concentrations of each treatment were 1, 2, 3, 4 and 5% in cell culture medium for 24, 48 and 72 h of incubation. Untreated cells, growing in culture medium, without any of concentration of juices, were used as a negative, untreated control. Cell proliferation was determined with Cell Proliferation ELISA, BrdU (Sigma-Aldrich, MO, USA), according to producing instruction.

### 2.7. Statistical analysis

The determination of bioactive compounds and antioxidant activity was carried out in triplicate each year of harvesting of vegetables. The level of individual polyphenols was determined in 2019. Results were expressed as the means ± standard deviation (SD). The results were expressed per dry weight (DW). Differences between content of analyzed compounds and antioxidant activity of young shoots and the mature vegetable were analyzed using Statistica v. 13.3 software (Tulsa, OK, USA). One-way analysis of variance ANOVA was conducted. Duncan's multiple range test was used for testing the differences. *P* values ≤ 0.05 were regarded as significant. Correlations between the tested compounds were examined using Pearson's correlation. The *in vitro* study of the influence of juice of vegetables on the proliferation of prostate and normal cancer cell lines was performed in three independent experiments and measured in triplicates. Shapiro–Wilk's test was applied to assess normality of distribution. The significance of differences, was assessed using: Student's *t*-test. *P* values ≤0.05 were regarded as significant.

## 3. Results

The content of bioactive compounds in dry weight (DW) of young 14-days shoots and red headed cabbage at full maturity significantly differed (*p* ≤ 0.05). In our previously publication we demonstrated a basic chemical composition of young shoots and the mature vegetable, including dry matter content.^10^ According to this data from cited paper, water content in young shoots was 92.7 g and in the mature vegetable was 91.5 g. This study indicated that dry matter content in juice of young shoots and of the mature vegetable was 5.0 and 7.2 g, respectively. Water content in juice of young shoots and the mature vegetable was 95 and 92.8 g, respectively. The results of our research showed that the vitamin C concentration in young shoots (795.9 mg/100 g DW) was about 2-fold higher than that of the mature cabbage (415.1 mg/100 g DW) (Table 1). The significantly larger (*p* ≤ 0.05) amount of vitamin C was determined in juice of young shoots (92.4 mg/100 mL of juice) than in juice of the mature vegetable (54.8 mg/100 mL of juice) (Table 3). The average content of total carotenoids in young shoots and the mature red headed cabbage was 123.3 and 22.2 mg/100 g DW, respectively (Table 1).

**Table 1:** Selected bioactive compounds contents in young shoots of red cabbage and the mature vegetable [mg/100 g DW]^a^

Vegetative form of plant	Vitamin C	Total carotenoids	Total polyphenols	Total anthocyanins
Young shoots	795.9^a^ ± 51.6	123.3^a^ ± 7.1	3201.5^a^ ± 208.5	336.7^a^ ± 27.7
Mature vegetable	415.1^b^ ± 31.3	22.2^b^ ± 4.7	3340.5^a^ ± 269.6	626.3^b^ ± 7.8

a DW dry weight; result are expressed as mean ± SD (*n* = 6); mean values with different letters (a and b) within the each column are statistically different (*p* ≤ 0.05).

Our study indicated that the level of total polyphenol content measured using the Folin–Ciocalteu reagent in young shoots and the mature vegetable was 3201.5 and 3340.5 mg/100 g DW, respectively and did not significantly differ (*p* > 0.05) (Table 1). In turn, the red headed cabbage had a statistically significant higher amount of total anthocyanins in comparison to young shoots – 626.3 and 345.1 mg/100 g DW, respectively (Table 1).

Phenolic acids and flavonoids were found in the analyzed young shoots, the mature vegetable and in juices obtained from them (Table 2, and 3). The following phenolic acids were determined: hydroxycinnamic acids (chlorogenic, caffeic, *p*-coumaric, ferulic, sinapinic acids) and hydroxybenzoic acids (gallic, 4-hydroxybenzoic, vanillic, syringic acids). Among flavonoids, flavanols (catechin, epicatechin), flavanones (naringin), flavonols (rutin, kaempferol, myricetin, isorhamnetin) and flavones (hesperidin, apigenin, luteolin) were found. The significantly largest amount of flavanols were determined in young shoots and juice obtained from them, while hydroxycinnamic acids were the most abundant polyphenols in red headed cabbage and in juice obtained from it. Young shoots and juice obtained from them were characterized by the highest content of catechin and epicatechin, whereas the mature vegetable and juice obtained from it was the richest in sinapinic acid and myricetin (Tables 2, 3, Fig. 4 and 5). Statistically significantly more vanillic acid, chlorogenic acid, catechin, epicatechin, naringin, rutin, kaempferol, isorhamnetin, hesperidin and apigenin was detected in young shoots. Syringic, *p*-coumaric, ferulic and sinapinic acids and myricetin were the dominant polyphenolic compounds found in the red headed cabbage. A similar amount (*p* > 0.05) of 4-hydroxybenzoic acid was found in both samples. Caffeic and gallic acids and luteolin were found only in young shoots (Table 2). Statistically significantly more vanillic, syringic, *p*-coumaric and ferulic acids, catechin, epicatechin, naringin, rutin, kaempferol and hesperidin was detected in juice of young shoots. 4-Hydroxybenzoic, chlorogenic and sinapinic acids and myricetin were the dominant polyphenolic compounds found in the juice of the red headed cabbage. Gallic and caffeic acids, kaempferol, isorhamnetin, luteolin and apigenin were found only in juice of young shoots (Table 3).

**Table 2:** Concentration of polyphenolic compounds in young shoots of red cabbage and the mature vegetable [mg/100 g DW]^a^

Polyphenolic compounds	Vegetative form of plant	
	Young shoots	Mature vegetable
Gallic acid	4.63 ± 0.01 a, C	ND
4-Hydroxybenzoic acid	12.73 ± 0.21 a, F	11.58 ± 0.69 a, AB
Vanillic acid	80.22 ± 0.41 a, L	5.12 ± 0.03 b, A
Syringic acid	8.77 ± 0.09 a, E	20.43 ± 0.13 b, BC
Chlorogenic acid	37.08 ± 0.07 a, J	26.69 ± 0.33 b, C
Caffeic acid	79.61 ± 0.47 a, L	ND
*p*-Coumaric acid	6.35 ± 0.04 a, D	7.38 ± 0.28 b, A
Ferulic acid	8.50 ± 0.14 a, E	12.55 ± 0.76 b, AB
Sinapinic acid	56.78 ± 0.01 a, K	497.55 ± 27.65 b, F
Catechin	216.26 ± 1.06 a, N	75.06 ± 2.08 b, D
Epicatechin	218.93 ± 1.55 a, O	73.81 ± 0.78 b, D
Naringin	24.79 ± 0.12 a, H	4.27 ± 0.21 b, A
Rutin	33.27 ± 2.06 a, I	4.88 ± 0.02 b, A
Kaempferol	3.93 ± 0.01 a, C	2.53 ± 0.07 b, A
Myricetin	142.75 ± 1.55 a, M	331.96 ± 18.91 b, E
Isorhamnetin	4.78 ± 0.01 a, C	2.91 ± 0.16 b, A
Hesperidin	17.41 ± 0.16 a, G	4.79 ± 0.09 b, A
Luteolin	2.17 ± 0.01 a, B	ND
Apigenin	0.82 ± 0.01 a, A	0.39 ± 0.01 b, A

a DW dry weight; result are expressed as mean ± SD (*n* = 3); mean values with different letters (a and b) within the individual polyphenolic compounds (row) are statistically different (*p* ≤ 0.05). Mean values with different letters (A–O) within the vegetative form of plant (column) are statistically different (*p* ≤ 0.05).

**Table 3:** Concentration of vitamin C and polyphenolic compounds in juices obtained from young shoots and the mature vegetable [mg/100 mL of juices]^a^

	Vegetative form of plant	
	Young shoots	Mature vegetable
**Bioactive compounds**		
Vitamin C	92.44 ± 3.75 a	54.84 ± 3.23 b

Polyphenols		
Gallic acid	0.44 ± 0.13 a, E	ND
4-Hydroxybenzoic acid	0.38 ± 0.213 a, D	2.31^a^ ± 0.12 b, F
Vanillic acid	0.99 ± 0.01 a, F	0.21^b^ ± 0.06 b, A
Syringic acid	1.59 ± 0.02 a, I	1.04^b^ ± 0.02 b, D
Chlorogenic acid	1.11 ± 0.01 a, G	2.58^b^ ± 0.73 b, G
Caffeic acid	1.13 ± 0.02 a, H	ND
*p*-Coumaric acid	1.10 ± 0.04 a, G	0.24^b^ ± 0.1 b, A
Ferulic acid	2.15 ± 0.1 a, K	1.44^b^ ± 0.42 b, E
Sinapinic acid	2.35 ± 0.01 a, L	9.63^b^ ± 0.12 b, J
Catechin	4.25 ± 0.04 a, P	1.48^b^ ± 0.62 b, E
Epicatechin	4.22 ± 0.04 a, O	3.71^b^ ± 1.17 b, H
Naringin	1.78 ± 0.02 a, J	0.61^b^ ± 0.02 b, B
Rutin	2.76 ± 0.04 a, M	0.42^b^ ± 0.05 b, C
Kaempferol	0.05 ± 0.01 a, B	ND
Myricetin	4.46 ± 0.09 a, R	9.05^b^ ± 0.3 b, I
Isorhamnetin	0.03 ± 0.01 a, A	ND
Hesperidin	4.18 ± 0.05 a, N	0.67^b^ ± 0.06 b, C
Luteolin	0.06 ± 0.01 a, C	ND
Apigenin	0.02 ± 0.01 a, A	ND

a Result are expressed as mean ± SD (*n* = 3); statistically significant differences (*p* ≤ 0.05) on the concentration of vitamin C and polyphenolic compounds within the each column are indicated by letter code a and b. Mean values with different letters (A–R) within the each row are statistically different (*p* < 0.05).

According to the three used assays: ABTS˙*, DPPH and FRAP, young shoots exhibited a statistically significant higher antioxidant activity in comparison to red headed cabbage (Table 4).

**Table 4:** Antioxidant activity of young shoots of red cabbage and the mature vegetable measured by ABTS, DPPH and FRAP assays [μmol Trolox/g DW]^a^

Vegetative form of plant	ABTS	DPPH	FRAP
Young shoots	700.1^a^ ± 29.2	186.7^a^ ± 14.8	324.9^a^ ± 6.8
Mature vegetable	347.0^b^ ± 33.2	123.4^b^ ± 15.8	240.4^b^ ± 25.2

a DW dry weight; result are expressed as mean ± SD (*n* = 6); mean values with different letters (a and b) within the each column are statistically different (*p* ≤ 0.05).

**Fig. 1:**
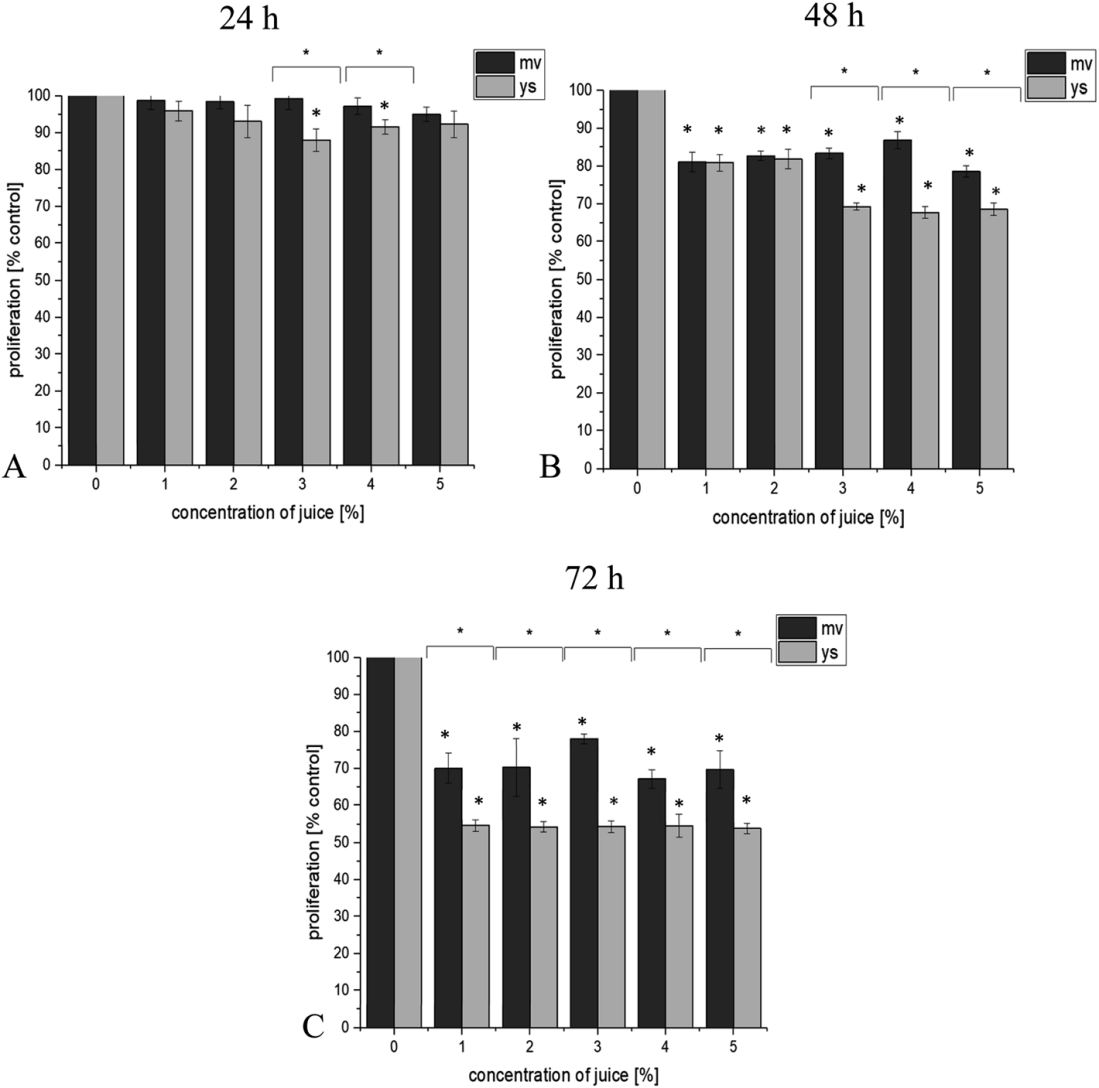
Effect of juice of the mature vegetable (mv) and of young shoots of red cabbage (ys) on DU145 prostate cancer cells proliferation in test with using Cell Proliferation ELISA, BrdU. The final concentrations of juices were 1, 2, 3, 4 and 5%. Effect of juices was measured after 24 h (A), 48 h (B) and 72 h (C) of incubation of cells. Values are expressed as means ± SD for *n* = 9, standardized to untreated control set as 100%. *p ≤ 0.05.

**Fig. 2:**
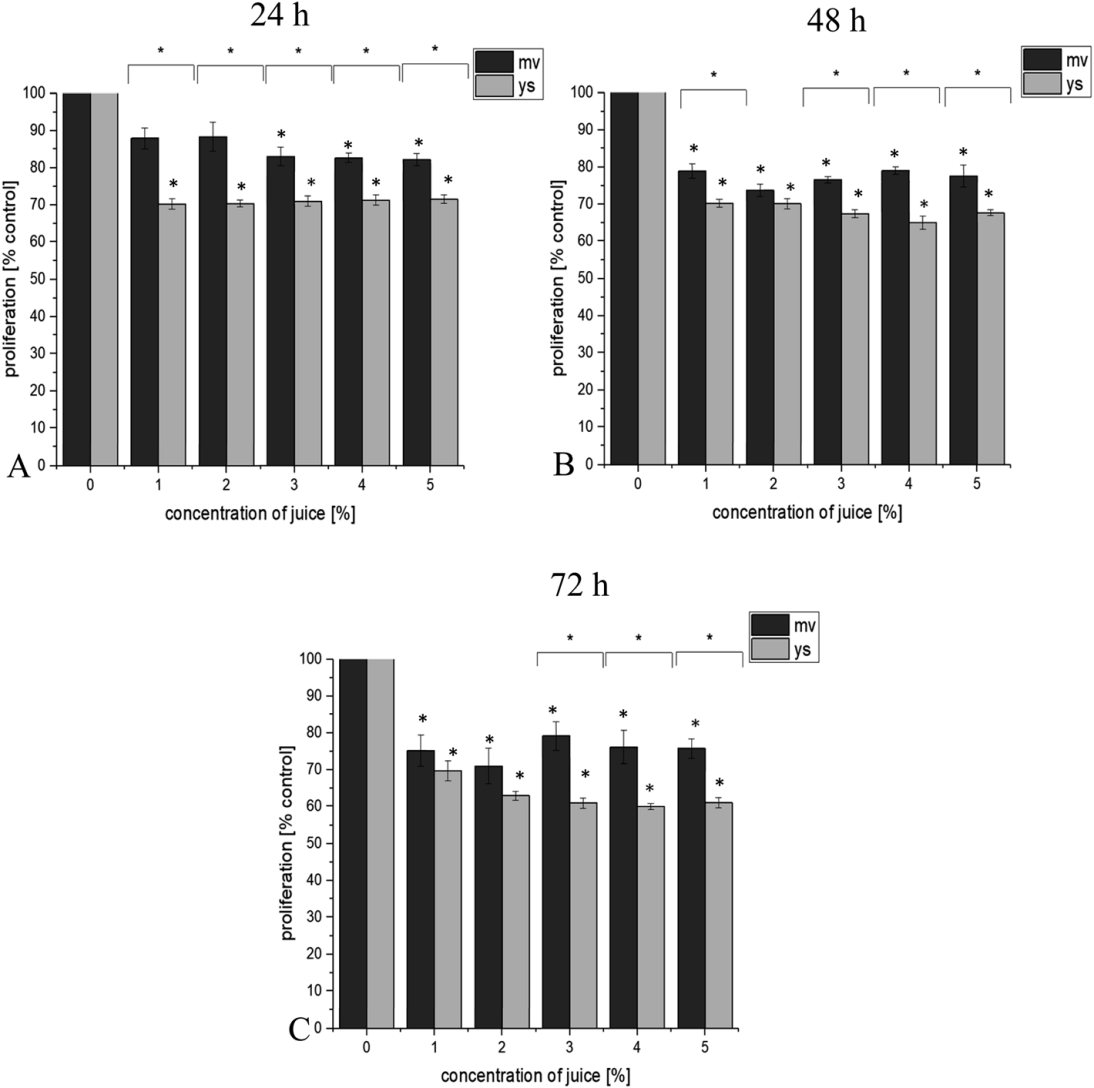
Effect of juice of the mature vegetable (mv) and of young shoots of red cabbage (ys) on LNCaP prostate cancer cells proliferation in test with using Cell Proliferation ELISA, BrdU. The final concentrations of juices were 1, 2, 3, 4 and 5%. Effect of juices was measured after 24 h (A), 48 h (B) and 72 h (C) of incubation of cells. Values are expressed as means ± SD for *n* = 9, standardized to untreated control set as 100%. *p ≤ 0.05.

**Fig. 3:**
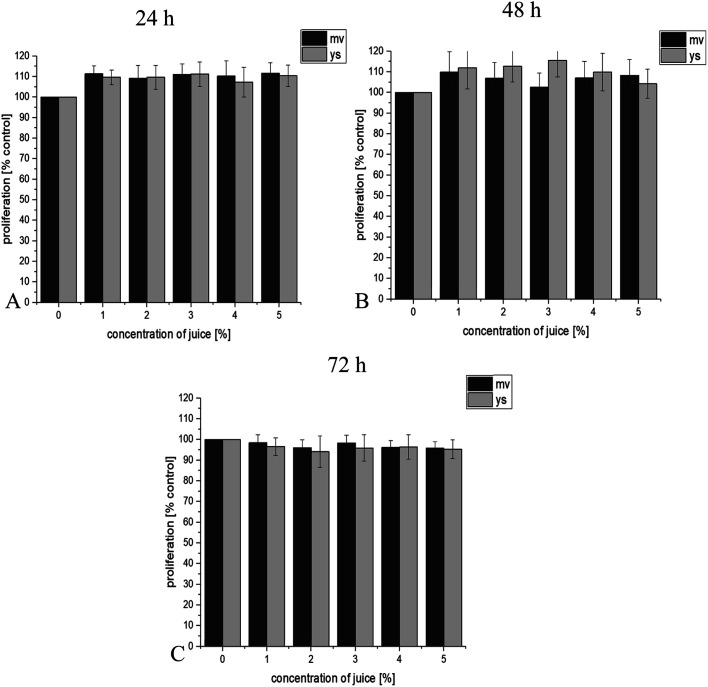
Effect of juice of the mature vegetable (mv) and of young shoots of red cabbage (ys) on PNT-2 prostate normal cells proliferation in test with using Cell Proliferation ELISA, BrdU. The final concentrations of juices were 1, 2, 3, 4 and 5%. Effect of juices was measured after 24 h (A), 48 h (B) and 72 h (C) of incubation of cells. Values are expressed as means ± SD for *n* = 9, standardized to untreated control as 100%. No statistical significance was demonstrated (*p* > 0.05).

BrDU labeling results showed that juice of young shoots and the vegetable at full maturity reduced the proliferation of studied cell lines in a dose and time dependent manner. Additionally, it has been observed that juice of young shoots effectively reduce proliferation of prostate cancer cell lines. 24 h incubation of DU145 cancer cells with 3 and 4% concentration of juice of young shoots significantly affected proliferation (*p* ≤ 0.05, Fig. 1A). Proliferation was reduced by approximately 12% at 24 h, 14–32% at 48 h and by 22–46% at 72 h (*p* ≤ 0.05, Fig. 1A–C). Prominent were results for LNCaP prostate cancer cells that showed a high level of susceptibility to all concentrations of juice of young shoots in our study. Statistically significant reduction in proliferation by 30% was observed already at 24 h, grown by 35% at 48 h and reached 40% at 72 h post-treatment (Fig. 2A–C). A reduction in proliferation was also observed after treatment LNCaP cells with juice of red headed cabbage, but this effect was significantly lower (by 18% at 24 h, 26% at 48 h and 29% at 7 2 h, Fig. 2A–C). More effective reduction in proliferation of LNCaP prostate cancer cell line treatment with the juice of young shoots, in comparison to juice of the mature vegetable, was observed already after 24 h of incubation. This effect was maintained during 48 and 72 h of incubation of cells with the tested juices (Fig. 2B and C). In the case of DU145 cells, this effect was observed in 24, 48 and 72 h of incubation for selected concentration of juices (Fig. 1A–C). Interestingly, PNT-2 normal prostate cells reacted other than cancer cells and did not show reduction in proliferation after treatment with juices (Fig. 3A–C).

**Fig. 4:**
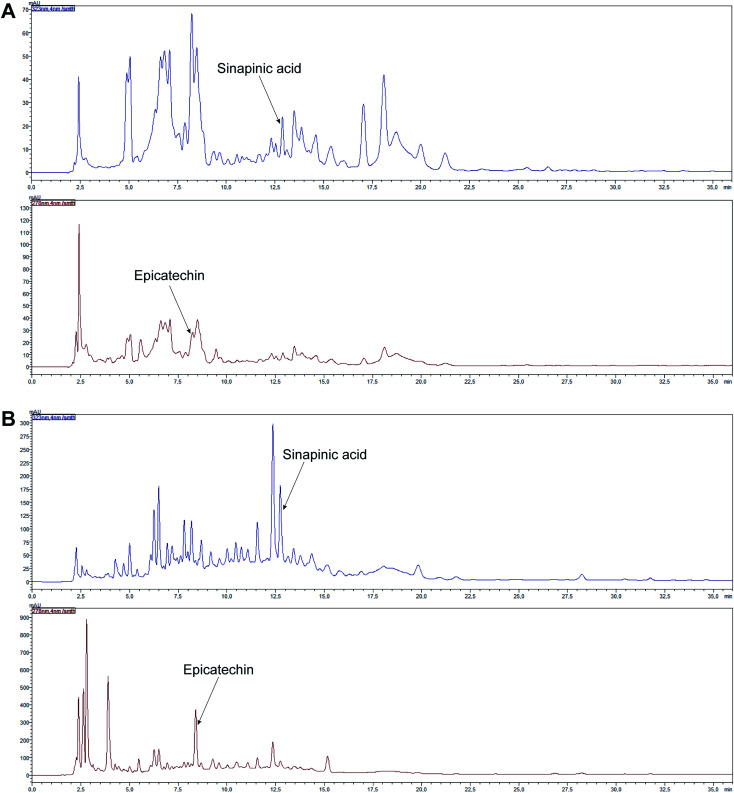
HPLC-DAD chromatograms of selected polyphenols in young shoots of red cabbage (A) and juice obtained from them (B).

**Fig. 5:**
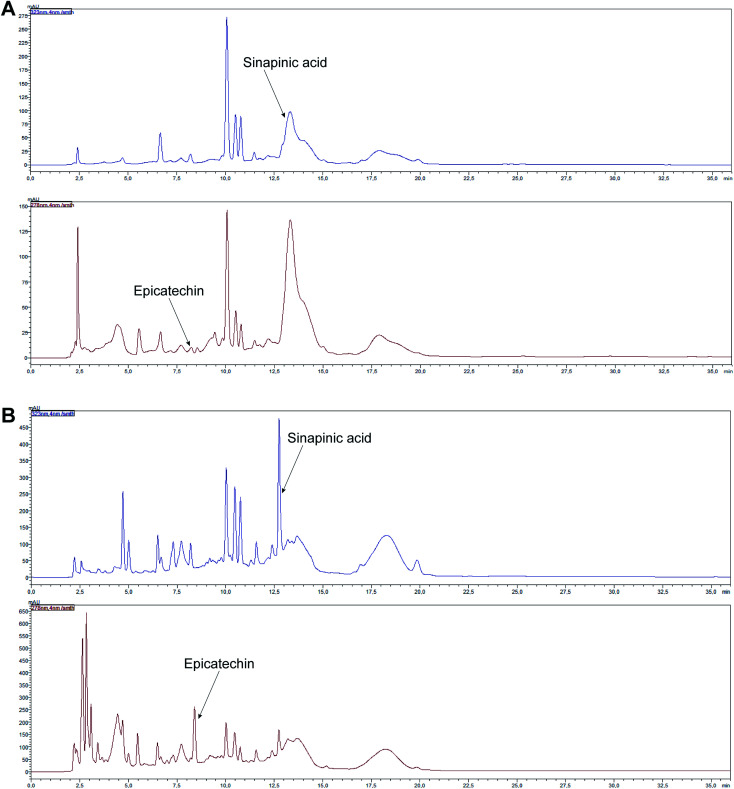
HPLC-DAD chromatograms of selected polyphenols in the red headed cabbage (A) and juice obtained from it (B).

## 4. Discussion

The consumption of Cruciferae in Poland and generally Eastern European countries has a long tradition. However, it seems important to study red cabbage seedlings, sprouts and young shoots as a vegetative parts of this popular vegetable. Due to their small size and biomass, very young sprouts may not be ideal for harvesting. Consequently, it was reasonable to study bigger ones – young shoots. In the present study, the major phytochemicals and their related antioxidant capacity in young shoots of red cabbage and the mature vegetable have been investigated. Additionally, the impact of the studied vegetable on the proliferation of prostate cancer cell lines *in vitro* has been analyzed.

### 4.1. Vitamin C in plant material and in juices

Thanks to its antioxidant properties, vitamin C decreases the adverse effect of free radicals and protects macromolecules which are implicated in cardiovascular diseases and some types of cancers.^15^ Vitamin C is involved in proliferation, differentiation and apoptosis of cancer cells. More than 85% of vitamin C in human diets is supplied by fruits, vegetables and herbs.^16^ The high concentration of vitamin C in young shoots obtained in our research can be associated with the fact that this compound has been directly implicated in the modulation of plant growth, including the early stage of germination of seeds.^17^ The content of vitamin C in young shoots was similar to that in the data published by Xiao *et al.*^18^ Šamec *et al.*, who evaluated 10 day white cabbage sprouts, also found a similar content of this antioxidant with respect to that reported in this study.^19^ The concentration of vitamin C in juice obtained from young shoots was higher than juice of the mature vegetable. Dumbravă *et al.* reported similar content of vitamin C in juice of the red headed cabbage.^20^

A similar content of vitamin C in red headed cabbage was shown in other works in comparison to the results obtained in the present study.^21,22^ Red cabbage growing in India was characterized by a lower concentration of vitamin C.^23^ Our results also do not correspond to the results published by Podsędek *et al.* who determined a higher vitamin C content.^24^

### 4.2. Total carotenoids in plant material

Carotenoids are the most predominant fat-soluble secondary plant metabolites. The most important feature of carotenoids is the antioxidant activity against reactive oxygen species and free radicals. Studies have shown that carotenoids have potent antitumor effects suggesting a potential preventive and therapeutic roles for these compounds. A higher dietary carotenoids intake has been correlated *e.g.* with a reduced cancer risk.^25^ The large amount of total carotenoids was found in young shoots and it is in line with the value reported by Xiao *et al.*^18^ However, Šamec *et al.* reported a lower content of carotenoids in white cabbage sprouts (37 mg/100 g DW).^19^

The results of Podsędek *et al.* research indicated that the content of this antioxidant in red headed cabbage was generally below 1 mg per 100 g FW of the vegetables tested (lower in comparison to our results for the mature vegetable).^26^ Our results also do not correspond to data published by Kaulmann *et al.* who reported a lower content of total carotenoids in red cabbage.^27^

### 4.3. Total polyphenols in plant material

Plant polyphenols are one of the largest and the most widespread specific group of secondary metabolites which play an important role in protecting against harmful effects of oxygen radicals and other highly reactive oxygen species. Literature data showed that polyphenol compounds are present in large amounts in seeds and during germination, reaching a several times higher value compared to plants at full maturity.^28^ Red cabbage sprouts characterized by Hunaefi *et al.* contained a higher level of total polyphenols compared to the young shoots which were analyzed in our study.^29^ Other authors who studied sprouts of red cabbage also found a higher level of total polyphenols.^9^ The content of these compounds in red cabbage sprouts was in the range of 5233 to 7497 mg/100 g DW (depending on germination time).

The results of the present study are generally in agreement with reports of Volden *et al.*^30^ and Wu *et al.*^31^ for red headed cabbage. Gaafar *et al.* showed that in red cabbage level of total polyphenols extracted with methanol was 2913 mg/100 g DW.^32^ Podsędek *et al.*, who studied the red headed cabbages var. Kissendrup and Koda, demonstrated a lower content of total polyphenols (1492.7 and 1293 mg/100 g DW, respectively).^26^ Other authors who studied the level of polyphenols in red headed cabbage reported lower values.^21,23,33,34^

### 4.4. Polyphenolic compounds in plant material and juices

The quantitative and qualitative composition of the polyphenol fraction determines the sensory quality of fresh plants. The color, palatability, external appearance or consistency of food products largely depends on the content of polyphenolic compounds. In our research, the HPLC analysis identified 19 polyphenolic compounds in young shoots, 16 in the vegetable at full maturity, 19 in juice of young shoots and 13 in juice of the mature vegetable. The profile of specific polyphenols differed between samples, flavanols dominated in young shoots and juice obtained from them and hydroxycinnamic acids dominated in red headed cabbage and juice obtained from it. In all samples, hydroxybenzoic acids, flavanones and flavones were the least numerous.

The flavonoids were the most predominant groups of polyphenols in the young shoots (69.3% of total) and juice obtained from them (66% of total). Phenolic acids accounted for the rest of the total polyphenols content. Among phenolic acids, caffeic, sinapinic, vanillic, syringic and ferulic acids turned out to be the most abundant compounds in young shoots and juice obtained from them. A similar profile of phenolic acids was found in 10 day red cabbage young sprouts (mainly caffeic, *p*-coumaric, chlorogenic, gallic and syringic acids).^29^ In our research, caffeic acid was found only in young shoots and juice obtained from them. It was noted that, among phenolic acids, caffeic acid occurred in large amounts in sprouts.^35^ Hydroxycinnamic acids are precursors of lignin biosynthesis. They are important in the first plant stages for rigidifying cell walls and rendering them impermeable to water. In our research, the hydroxycinnamic acids constituted a second group of identified polyphenols in young shoots and juice obtained from them (19.6% and 23.7%, respectively). Our results do not correspond with Soengas *et al.* who showed that hydroxycinnamic acids are predominant polyphenols and flavanols were found in lower concentration in ted cabbage sprouts.^36^ With respect to the hydroxycinnamic acids profile, young shoots of red cabbage and juice obtained from them were composed mostly of caffeic, sinapinic, ferulic and chlorogenic acids and a smaller portion of the total amount of these compounds were other hydroxycinnamic acids. Soengas *et al.* examined that young sprouts of red cabbage had a high content of sinapinic acid.^36^ Francisco *et al.* indicated that the polyphenol profile of cruciferous sprouts is composed mostly of hydroxycinnamic acids (sinapinic, chlorogenic, *p*-coumaric and ferulic acids) and flavonols (mainly kaempferol and isorhamnetin).^37^ Flavonoids are described as the most common polyphenolic compounds of several *Brassica* vegetables.^7^ Our research indicated that young shoots of red cabbage and juice obtained from them had a higher content of these polyphenols in comparison to the mature vegetable and juice obtained from it. In our research it was found that the flavanols (catechin and epicatechin) are a major group of polyphenols present in young shoots and juice obtained from them. There is a little information on the content of these compounds in *Brassica* vegetables, including young shoots or sprouts. Seedling of white cabbage contained a higher content of catechin in comparison to our study on young shoots of red cabbage.^38^ Due to the numerous health promoting properties of catechins, it is recommended that products containing them should be included into the daily diet. Research results indicate that catechins can regulate the growth of cancer cells by interacting with epidermal growth factor (EGF).^39^ Flavonols, that in the case of tested plants embraced mainly myricetin, but also rutin, kaempferol and isorhamnetin, are considered to be antioxidants compounds with antiinflammatory and anticancer properties.^40^ Among these polyphenols, kaempferol and isorhamnetin were identified in young sprouts of red cabbage by Soengas *et al.*^36^ We observed a higher content of flavones in young shoots than in the vegetable at full maturity. No flavones have been identified in the juice of the mature vegetable. The presence of luteolin was noted only in young shoots and juice obtained from them. It is known that a high intake of apigenin (which we found in young shoots and juice obtained from them) can be inversely associated with the risk of cancer.^41^

Phenolic acids constituted 53.7% of the total described polyphenols in red headed cabbage and 52.2% in juice obtained from it. The main polyphenolic compounds identified in the mature vegetable and in juice obtained from it were sinapinic acid and myricetin. Other polyphenols-catechin, epicatechin and chlorogenic acid were also identified in large amount. Phenolic acids and flavanols were also noted to be the major compounds in red headed cabbage by Buss Cruz *et al.*^42^ The study carried out by Park *et al.* revealed a profile in red cabbage methanolic extract with the predominance of sinapinic and *p*-coumaric acids and kaempferol and quercetin.^43^ Sinapinic acid is typically found in Brassicaceae species and was the main compound identified in red headed cabbage.^44^ Mazzucotelli *et al.*^33^ and Mattila and Hellström^45^ reported a higher content of this compound in comparison to our results. Sinapinic acid and its derivatives have various biological activities. For example, Yuan *et al.*^46^ demonstrated that sinapinic acid exerts anti-inflammatory and antiedema effects. *p*-coumaric acid and ferulic acid were hydroxycinnamic acid derivatives identified in our samples. Red headed cabbage had a higher content of these polyphenols in comparison to young shoots. *p*-coumaric and ferulic acids have also been found in cabbage (no mention of variety).^43^ Mazzucotelli *et al.*^33^ reported a higher content of these compounds in comparison to our results. Mattila and Hellström^45^ found a smaller amount of vanillic and 4-hydroxybenzoic acids in comparison to our study and no presence of syringic acid in red headed cabbage. Koss-Mikołajczyk *et al.* who studied the polyphenol profile of red cabbage showed a lower content of chlorogenic acid in comparison to our results.^47^ In our research, the most abundant flavonoids in red cabbage were flavonols: myricetin, rutin, isorhamnetin and kaempferol. Miean and Mohamed reported that the only flavonol found in cabbage was myricetin (lower content in comparison to our results).^48^

### 4.5. Total anthocyanins in plant material

Similar to other natural polyphenolic compounds found in plants, anthocyanins have a strong antioxidant effect. Health benefits of anthocyanins include antiproliferative, anticancer, antiviral, anti-bacterial and anti-allergic activates.^49^ Red cabbage seedlings develop rapidly and produce large amounts of anthocyanins.^50^ 4 Day-old red cabbage seedlings grown under light conditions contained a similar quantity of anthocyanins in comparison with our results.^51^ A lower content of anthocyanins in 10 day-old sprouts and in 7 day seedlings of red cabbage was reported by Hunaefi *et al.*^29^ and Yuan *et al.*,^46^ respectively.

Red headed cabbage, which was also confirmed in our research, is a good source of anthocyanins.^52^ The concentration of total anthocyanins in red headed cabbage was studied by many authors. Depending on the analyzed part of red cabbage, total content of anthocyanins ranged from 544 to 696 mg/100 g DW.^43^ The working groups of Leja *et al.*^53^ and Podsędek *et al.*^26^ reported a similar content of total anthocyanins in red headed cabbage var. Haco, Koda and Kissendrup in comparison with our results. Buss Cruz *et al.*^42^ and Sankhari *et al.*^54^ also found a similar value of total anthocyanin in red cabbage extract. Our results were higher than that in data published by Bernstein and Noreña who reported that anthocyanins levels in red cabbage were as follows: 261 mg/100 g DW.^55^ Other authors obtained a higher concentration of anthocyanins with respect to that reported in this study.^56,57^

Biosynthesis of anthocyanins in plant tissues is controlled by the transcript level of the corresponding genes and the accumulation of these substances is affected by various environmental conditions.^51,58^ A characteristic, commonly observed phenomenon is the biosynthesis of anthocyanins in young seedlings and leaves in which the chlorophyll content is low. An insufficiently developed photosynthetic system means that tissues are able to absorb only a small part of the light available. Increased biosynthesis and anthocyanin accumulation at that time indicate their role in protecting young tissue against adverse environmental conditions.^59^ This phenomenon may explain the presence of anthocyanins in young shoots of red cabbage.

### 4.6. Antioxidant capacity of plant material

In the present study, the antioxidant activity of plant samples was determined using three methods: with ABTS˙*, DPPH and FRAP reagents. ABTS˙* presented higher antiradical activity than the DPPH and FRAP, which was also described by Kusznierewicz *et al.*^60^ The used methods presented coherent results for the studied material – young shoots of red cabbage showed a higher radical scavenging activity in comparison to red headed cabbage. Baenas *et al.* who studied the 4 day red cabbage sprouts, demonstrated a similar radical scavenging activity in DPPH and FRAP assays.^8^ 7 Day red cabbage sprouts showed a high antioxidant activity in FRAP and DPPH assay, but the results were lower in comparison to our data.^36^

The high antioxidant activity of the red headed cabbage was confirmed by Proteggente *et al.*^21^ Kaulmann *et al.* who analyzed the antioxidant activity of the mature red cabbage using the FRAP method reported similar results in comparison to those presented in this study.^27^ Working groups of Biesiada *et al.*^56^ and Podsędek *et al.*^26^ who evaluated the radical scavenging activity of red headed cabbages var. Kissendrup and Koda showed a lower values in ABTS˙* and DPPH tests in comparison to our result. Results of our research indicated, that antioxidant activity of red cabbage decreases with time of cultivation. This suggests that red cabbage could have the highest antioxidant activity when the leaves are young.

The discrepancies between the quantity of biologically active compounds and variation in the antioxidant activity of vegetable samples studied and that reported by other authors can be caused by many factors. The differences between content of vitamin C, carotenoids and polyphenols might result from natural variation of tested material, time of harvest and ripening state, cultivation and environmental conditions, as well as condition of post-harvest storage.^56,61,62^

### 4.7. Correlation

In order to evaluate the relationship between the content of bioactive compounds and the antioxidant activity expressed by the different assays performed, a Pearson's correlation coefficient was analyzed and some significant correlations were found (Table 5). In the young shoots and the mature vegetable, the strong dependency between antioxidant capacity and total polyphenols was found. Furthermore, in both analyzed samples, the antioxidant activity strongly depended on vitamin C content. Results obtained by Baenas *et al.*^8^ and Soengas *et al.*^36^ confirm strong correlation between values of total polyphenols and antioxidant capacity in red cabbage sprouts. Biesiada *et al.* determined antioxidant properties of red headed cabbage and proved a positive correlation between polyphenols content and antioxidant activity.^56^ Hounsome *et al.* showed that antioxidant activity in *Brassica* species was correlated with vitamin C and total polyphenols content.^63^ In our investigation it was possible to confirm high antioxidant activity of young shoots at considerably elevated level of carotenoids. Lipid-soluble antioxidants were responsible for up to 20% of the total antioxidant activity of *Brassica* vegetables.^64^ In a study of Ismail *et al.*^65^ relationship between antioxidant activity and total polyphenol content in cabbage was not found, although cabbage was characterized by a high antioxidant activity. The findings of this study could indicate that each type of vegetable had a different antioxidant activity which was connected with a variety of antioxidant components, not only polyphenols.

**Table 5:** Correlation matrix of bioactive compounds content with antioxidant activity measured ABTS, DPPH and FRAP assays in young shoots of red cabbage and the mature vegetable

	Vitamin C	Total carotenoids	Total polyphenols	Total anthocyanins	ABTS	DPPH	FRAP
**Young shoots**							
Vitamin C	1.0000	—	—	—	—	—	—
Total carotenoids	0.6097	1.0000	—	—	—	—	—
Total polyphenols	0.5936	0.7392	1.0000	—	—	—	—
Total anthocyanins	0.4238	0.3904	0.4342	1.0000	—	—	—
ABTS	0.9078	0.8195	0.8228	0.7903	1.0000	—	—
DPPH	0.6927	0.9287	0.8913	0.5248	0.8860	1.0000	—
FRAP	0.4440	0.7480	0.8956	0.2683	0.6604	0.9057	1.0000

**Mature vegetable**							
Vitamin C	1.0000	—	—	—	—	—	—
Total carotenoids	0.4010	1.0000	—	—	—	—	—
Total polyphenols	0.7550	0.6716	1.0000	—	—	—	—
Total anthocyanins	0.8205	0.7401	0.6355	1.0000	—	—	—
ABTS	0.9729	0.2774	0.6819	0.7103	1.0000	—	—
DPPH	0.9566	0.3803	0.8666	0.6896	0.9345	1.0000	—
FRAP	0.8150	0.0756	0.3405	0.7010	0.7413	0.6965	1.0000

### 4.8. Cell proliferation

Oxidative stress is recognized as an important factor associated with the development of cancer. During a chronic inflammatory process in the body, the number of free radicals rapidly increases, and they have the ability to damage cell membranes, DNA and proteins. This damage lead to disturbances in biological functions of cells, which in turn causes abnormalities in their metabolism. Antioxidants prevent the formation of free radicals, take part in their neutralization as well remove oxidative damage. Numerous studies showing that consumption of cruciferous vegetables is consistently associated with strong protective effects against specific cancers. The most characteristic compounds of cruciferous vegetables with identified anticancer properties are glucosinolates. Mechanical and thermal damage to the cells leads to the activation of the enzyme myrosinase, which causes hydrolysis of glucosinolates to active compounds of disintegration. These compounds are strong associated with potential chemopreventive effects targeting multiple biological pathways by modulating cellular events leading to apoptotic cell death.^3^ In our previously publication we have shown that young shoots are a better source of these anticancer compounds than the mature vegetable.^10^ Red cabbage contains numerous other reactive phytochemicals that inhibit several key events involved in tumor cell growth and proliferation (studied vitamin C, carotenoids and polyphenols). In this study we determined higher amount of vitamin C in juice of young shoots than juice of the mature vegetable. The total content of polyphenolic compounds was similar in both juices. It is common knowledge that the antioxidant and anticancer properties of vegetables result from the synergistic effects of several phytochemicals present in these foods.^66^ Vitamin C has an antiproliferative activity which has been observed *in vitro* as well *in vivo* and likely results from the inhibition of expression of genes involved in protein synthesis.^67^ Numerous phenolic compounds have been investigated for their potential use in chemopreventive. By modulating cell signaling pathways, polyphenols activate cell death signals and induce apoptosis in cancer cells resulting in the inhibition of cancer development or progression.^68^ Roy *et al.* have shown that that commonly consumed cruciferous vegetables (including cabbage) contain water-soluble components that possess antioxidant activity and antiproliferative activity against the HL-60 cell line *in vitro*.^69^ Our results showed that juice (mix of bioactive phytochemicals) of young shoots and the mature vegetable reduce the proliferation of prostate cancer cell lines DU145 and LNCaP, without having an effect on the proliferation of normal prostate cancer cell line PNT-2. Additionally, it has been observed that juice of young shoots more effectively decreased the proliferation of prostate cancer cell lines than juice of the mature vegetable. This effect could be the results of high amount of bioactive compounds, including glucosinolates (described previously, as mentioned above), polyphenolic compounds, vitamin C and carotenoids in young shoots of red cabbage. Kestwal *et al.* showed that the aqueous extracts of cruciferous sprouts inhibit proliferation of HepG2 hepatoma cells and colon cancer CT26 cells in a dose dependent manner.^70^ Results obtained by Boivin *et al.* indicated that cruciferous vegetables, including red cabbage had potent inhibitory activities against most studied cancer cell lines (also on the prostate cancer cell line PC-3).^71^ The bioactivity of red headed cabbage juice on colon cancer cell line HCT-116 were analyzed by Kannan.^72^ Results of this research indicate that the studied juice reduced HCT-116 cell proliferation.

## 5. Conclusions

Owing to the fact that not all vegetables have the same bioactive phytochemicals composition, and that not all phytochemicals have the same antioxidant capacity, it is important to recognize which vegetables have the highest antioxidant capacity and introduce them into the daily diet. Based on our analysis, it can be suggested that young shoots of red cabbage could be a better source of vitamin C and carotenoids in comparison to the mature vegetable. Additionally, young shoots were characterized by a similar level of total polyphenols. Among polyphenols, flavonoids were predominant in young shoots, while phenolic acids in the mature vegetable. The presence of anthocyanins in young shoots gives an added value to these products. Young shoots were characterized by the highest antioxidant activity. Moreover, we determined a high amount of vitamin C and polyphenolic compounds in juice of young shoots. The results of our *in vitro* studies have shown that the juice of young shoots of red cabbage effectively reduced the proliferation of cancer cells, more than the juice of the mature vegetable. The increased consumption of the studied vegetables could play an important role in chemoprevention. Future research should be focused on the influence of bioactive compounds found in young shoots of red cabbage on the mechanism of prostate cancer cell death *in vitro*.

## Conflicts of interest

There are no conflicts to declare.

## Supplementary Material
